# Longitudinal assessment of chest computerized tomography and oxygen saturation for patients with COVID-19

**DOI:** 10.1186/s43055-020-00376-y

**Published:** 2020-12-09

**Authors:** Ahmed M. Osman, Suzan Farouk, Nehad M. Osman, Ahmed M. Abdrabou

**Affiliations:** 1 Radiology Department, Faculty of Medicine, Ain Shams University, Ramses street, Cairo, 11591 Egypt; 2 Chest Disease Department, Faculty of Medicine, Ain Shams University, Cairo, Egypt

**Keywords:** Coronavirus, Computed tomography (CT), Ground-glass opacity (GGO), Oxygen saturation, Severity score

## Abstract

**Background:**

COVID-19 is a pandemic disease and is important to know the nature of the disease during follow-up. We aimed to study different imaging signs and changes that occurred during the initial scan, follow-up, and complications. Moreover, to study the CT severity score and its relation to the patients’ clinical condition using oxygen saturation as a parameter. This was a retrospective study conducted on 125 patients, including 293 CT studies, from March till the end of August 2020. The mean age was 47.4 ± 15.7 years and 64.8% of the patients were males. All patients proved to have COVID-19 by the RT-PCR test. The CT studies of the patients were divided into four stages according to the timing after the onset of symptoms. The incidence of different CT features, patterns, complications, CT severity score, and oxygen saturation were recorded in different stages.

**Results:**

During follow-up studies, GGOs were the most constant and common CT features. Consolidation and crazy paving showed gradual progression to reach the peak at the 3rd stage. Mixed attenuation pattern was the commonest pattern at the 3rd stage while a pure GGO pattern was the commonest feature in other stages. The complications occurred mostly in the 3rd stage. Nevertheless, the CT severity score showed an inverse relation with oxygen saturation.

**Conclusion:**

Radiological evaluation of COVID-19 pneumonia showed gradual progression till the peak critical stage at 8-14 days from the onset of symptoms. Consolidation and mixed attenuation pattern can be considered as CT signs of disease severity.

## Background

Novel COVID-19 virus is a newly discovered strain of the coronaviruses, which include six well-known strains. It is first described in Wuhan, China, in December 2019 [1, 2].

The novel COVID-19 virus is transmitted via respiratory droplets, as well as physical contact. The estimated incubation period is from 3 to 7 days, and some reported up to 14 days [3, 4].

The pathogenesis of the novel COVID-19 virus is still a matter of debate. Some authors explained the effect of this virus via the use of respiratory angiotensin-converting enzyme 2 (ACE 2) which causes pulmonary interstitial followed by parenchymal damage. Consequently, these changes are reflected in CT images differently according to the stage of the disease [5].

While others highlighted the thrombotic effect of the virus inside the micro-circulation of the lung causing an extensive widespread pulmonary embolism (PE). The myocardial injury was also reported in some cases with high mortality incidence [6].

Recent studies underlined the role of computed tomography (CT) in the detection, diagnosis, and monitoring of the progression of cases with novel COVID-19 infection as well as the assessment of the therapeutic response. The typical CT features of cases with COVID 19 pneumonia include bilateral, multifocal, peripheral, and ground-glass opacities (GGOs) with sub-segmental patchy consolidations that mostly involve the posterior segments and both lower lobes [7–9].

Understanding the radiological changes that may occur over time via serial CT chest exams, during the period of COVID-19 infection, may improve the diagnosis and management [10]. Most of the COVID-19 studies found the most severe features and CT abnormalities within 10 days after the onset of symptoms [11].

This study aimed at assessing the radiological changes that occurred during COVID-19 infection and the incidence of complications. Also, we assessed the relation between CT severity of the disease and the patient’s oxygen saturation.

## Methods

### Patients

One hundred twenty-five patients known to have novel COVID-19 infection by positive reverse transcription-polymerase chain reaction (RT-PCR) were enrolled in this retrospective study. All patients attended the hospital from March 2020 to the end of August 2020 and underwent high-resolution CT chest without contrast on admission, followed by at least one follow-up CT within 3 weeks from the onset of symptoms. The study was approved by the ethical committee of our institute and the patients’ consents were waived being a retrospective study after insuring special instruction regarding complete security and confidentiality of the patients’ data.

### Inclusion criteria

All patients included in this study were positive by RT-PCR for COVID-19 infection and had CT on admission as well as another follow-up CT or more within 3 weeks from the onset of symptoms.

### Exclusion criteria

Non-available PCR results. Unavailable data about the patient’s oxygen saturation. Patients under the age of 18 years.

### CT technique

- The study was performed using an 80-slice CT machine (Prime Aquilion, Toshiba, USA). The patients were instructed to lie in a supine position and to elevate their arms behind their heads to avoid bony artifacts and to hold their breaths during the scanning time. Scouts were taken starting from 1 cm above the lung apices down to 1 cm below the lowest costo-phrenic angle. Parameters for the acquisition included tube voltage of 120 kV, milliampere ranged between 150 and 400 mA according to the patient’s weight, 1.25 mm slice thickness, 0.625 mm slice interval, 512 × 512 matrix, and tube speed 35 mm/rotation (0.5 s rotation time).
- Image processing and interpretation: The images were transferred to the workstation (Fuji Synapse workstation or Paxera-Ultima workstation) for reviewing the axial cuts and multi-planar reformation. The image analysis was done in consensus by two radiologists experienced in chest imaging with at least 5 years’ experience blinded to the timing of the CT and clinical condition of the patients. The analysis was carried out with an inter-observer agreement. The following items must be fulfilled during image interpretation:
  - CT features assessment: Presence of GGO, consolidation, crazy paving, and fibrosis.
  - CT pattern assessment:
    - Negative pattern: no evidence of pneumonia.
    - Pure GGO pattern: CT with GGO and/or crazy paving and no other CT features.
    - Consolidation pattern: CT with consolidation patches and no other CT features.
    - Mixed attenuation pattern: CT with GGO mixed with consolidation which was subdivided according to the predominant features into mixed with predominant GGO or mixed with predominant consolidation.
  - The number of lobes involved in each time during follow-up.
  - Assessment of the severity progression:
    - Each of the five lung lobes was visually scored on a scale of 0 to 5. Score 0 indicates no involvement, score 1 if less than 5% involvement; score 2 if 5–25% involvement; score 3 if 26–49% involvement; score 4 if 50–75% involvement; and score 5 if more than 75% involvement. The total CT score was the sum of the individual lobar scores and ranged from 0 (no involvement) to 25 (maximum involvement).

The CT of all selected patients are divided into 4 stages according to the date of CT after the onset of symptoms: 1st stage (early stage) = 0-3 days, 2nd = 4-7 days, 3rd = 8-14 days, and 4th (late stage) = 15-21 days.

### Pulse oximetry

The oxygen saturation SpO_2_ of all included patients was recorded within 24 h before or after every CT. Oxygen saturation < 93% was considered as an indication for oxygen supply [12].

### Recording of the complications

The complications that occurred during follow-up of the patients were recorded at the time of incidence, for example, pleural effusion, pulmonary embolism, and the need for oxygen supply.

### Sample size calculation

Using Epi Info 7 Program for sample size calculation and according to Wang et al. [10], the expected proportion of patients with residual CT abnormality on discharge = 94%. Setting a margin of error at 5% and at a 95% confidence level, the sample size of at least 90 patients will be needed.

### Statistical method

The collected data were coded, tabulated, and statistically analyzed using IBM SPSS statistics (Statistical Package for Social Sciences) software version 18.0, IBM Corp., Chicago, USA, 2009.

Descriptive statistics were done for quantitative data as minimum and maximum of the range as well as mean ± SD (standard deviation) for quantitative normally distributed data, while it was done for qualitative data as number and percentage. Inferential analyses were done for quantitative variables using the ANOVA test. In qualitative data, inferential analyses for independent variables were done using the chi-square test for differences between proportions and Fisher’s exact test for variables with small expected numbers. Post hoc Bonferroni test for multiple comparisons. The level of significance was taken at *P* value < 0.050 is significant; otherwise, it is non-significant.

## Results

**Fig. 1:**
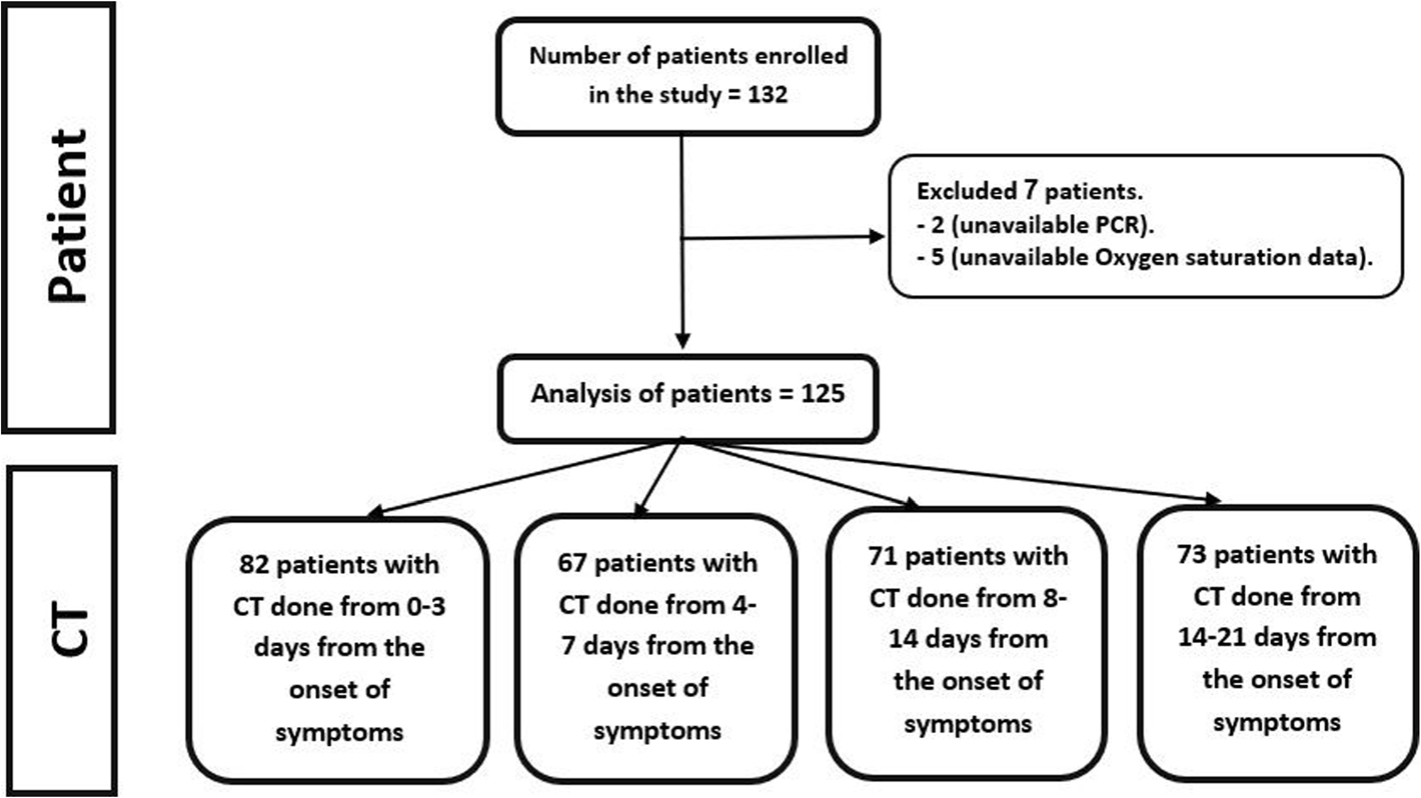
CONSORT flow diagram showing the number of patients at each phase of the study

This is a retrospective cohort observational cross-sectional study conducted on 125 patients known to have COVID-19 infection as proved by RT-PCR after the exclusion of 7 cases due to unavailable RT-PCR results or unavailable data about the oxygen saturation (Fig. 1).

### Demographic data

The mean age of the patients was 47.4 ± 15.7 years ranging from 19 to 85 years. Eighty-one patients were males. The main presenting symptoms for the selected patients were dyspnea, recorded in 66.4% of cases, followed by the fever which was observed in 55.2% of patients. The patients underwent CT chest at admission and at least one follow-up CT, giving a total of 293 CT studies (average 2.34 CT/patient) (Table 1).

**Table 1:** Demographic data of the selected patients including age, sex, presenting complaint, and average CT studies

**Variables**	**Mean ± SD**	**Range**
**Age (years)**	47.4 ± 15.7	19.0–85.0
	**Number**	**%**
**Gender**		
**Male**	81	64.8
**Female**	44	35.2
**Presenting complain**		
**Dyspnea**	83	66.4
**Fever**	69	55.2
**Cough**	52	41.6
**History of contact to diseased person**	14	11.2
**GIT symptoms**	5	4.0
**Respiratory distress**	3	2.4
**CT number**		
**2**	87	69.6
**3**	33	26.4
**4**	5	4.0

### Longitudinal assessment of the CT features

**Fig. 2:**
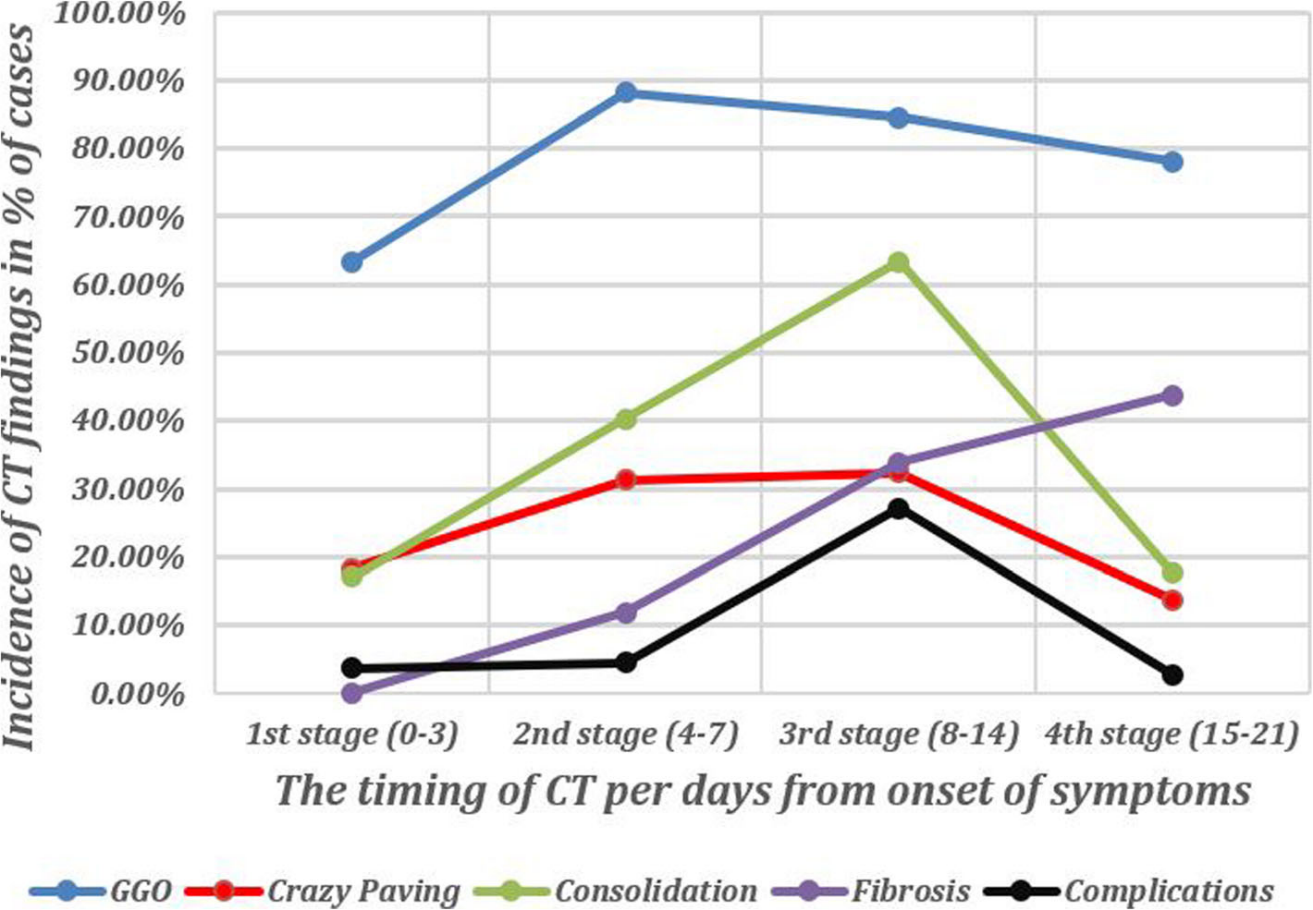
A diagram showing the changes in the incidence of different CT findings and the incidence of complications during different stages of follow-up of cases with COVID-19 infection

*GGO* was the commonest features found in all stages of the disease during follow-up. Yet, it showed relative low incidence during the early stage (1st stage = 63.4%) then increased to reach the peak at 2nd stage (88.1%), then decreased gradually in the 3rd and 4th stages (84.5% and 78.1% respectively) but remained higher than the 1st stage (Table 2) (Fig. 2).

**Table 2:** The CT changes, CT pattern, CT severity score, complications, and oxygen saturation that occurred during different stage of the COVID-19 course

Findings	1st stage (CT 0-3 days)	2nd stage (CT 4-7 days)	3rd stage (CT 8-14 days)	4th stage (CT 15-21 days)	***P*** value
**Number of the patients**	82	**67**	71	73	
**GGO**	52 (63.4%) a	**59 (88.1%) b**	60 (84.5%) b	57 (78.1%) ab	#0.001*
**Crazy paving**	15 (18.3%) ab	21 (31.3%) ab	**23 (32.4%) a**	10 (13.7%) b	#0.014*
**Consolidation**	14 (17.1%) a	27 (40.3%) b	**45 (63.4%) c**	13 (17.8%) a	# < 0.001*
**Fibrosis**	0 (0.0%) a	8 (11.9%) b	24 (33.8%) c	**32 (43.8%) c**	# < 0.001*
**CT Pattern**					
**Pure GGO**	39 (47.6%) abc	34 (50.7%) c	20 (28.2%) b	**46 (63.0%) ac**	§ < 0.001*
**Consolidation**	1 (1.2%) a	1 (1.5%) a	**5 (7.0%) a**	2 (2.7%) a	
**Mixed**	13 (15.9%) a	25 (37.3%) b	**40 (56.3%) b**	11 (15.1%) a	
**Negative**	**29 (35.4%) a**	7 (10.4%) b	6 (8.5%) b	14 (19.2%) ab	
**Mixed pattern**					
**Predominant GGO**	6 (46.2%)	**13 (52.0%)**	9 (22.5%)	5 (45.5%)	§0.069
**Predominant Consolidation**	7 (53.8%)	12 (48.0%)	**31 (77.5%)**	6 (54.5%)	
**Complications**	3 (3.7%) a	3 (4.5%) a	**19 (27.1%) b**	3 (4.1%) a	# < 0.001*
**Types of complications**					
**Hypoxia**	3 (3.7%) ab	3 (4.5%) ab	**11 (15.7%) a**	1 (1.4%) b	§0.004*
**Pl. effusion**	0 (0.0%) a	0 (0.0%) a	**13 (18.6%) b**	2 (2.7%) a	§ < 0.001*
**Resp. distress**	0 (0.0%)	0 (0.0%)	**2 (2.9%)**	0 (0.0%)	§0.105
**Pneumothorax**	0 (0.0%)	0 (0.0%)	**1 (1.4%)**	0 (0.0%)	§0.469
**Number of the lobes affected**	2.0 ± 1.9 a	3.3 ± 1.7 bc	**3.8 ± 1.6 c**	2.7 ± 1.8 ab	^< 0.001*
**CT severity score**	2.4 ± 3.0 a	5.2 ± 3.8 b	**7.1 ± 5.1 c**	3.7±3.5 ab	^< 0.001*
**Oxygen saturation SpO**_**2**_ **%**	96.8 ± 2.2 a	96.3 ± 2.0 a	**93.9 ± 3.7 b**	96.6±1.9 a	^< 0.001*

Post hoc Bonferroni test (homogenous groups had the same symbol “a, b, c”)

^ANOVA test

#Chi square test

§Fisher’s exact test

*Significant

*Consolidation and crazy paving* were lowest at 1st stage then frequently accelerated to reach the peak at the 3rd stage (63.4% for the consolidation and 32.4% for the crazy paving), then declined in the 4th stage to become lower than seen in the early 1st stage (Table 2) (Fig. 2).

*Fibrosis* was absent at the early 1st stage and constantly grew up along the course of the disease to peak at the late 4th stage (43.8%) (Table 2) (Fig. 2).

### Longitudinal assessment of the CT pattern

**Fig. 3:**
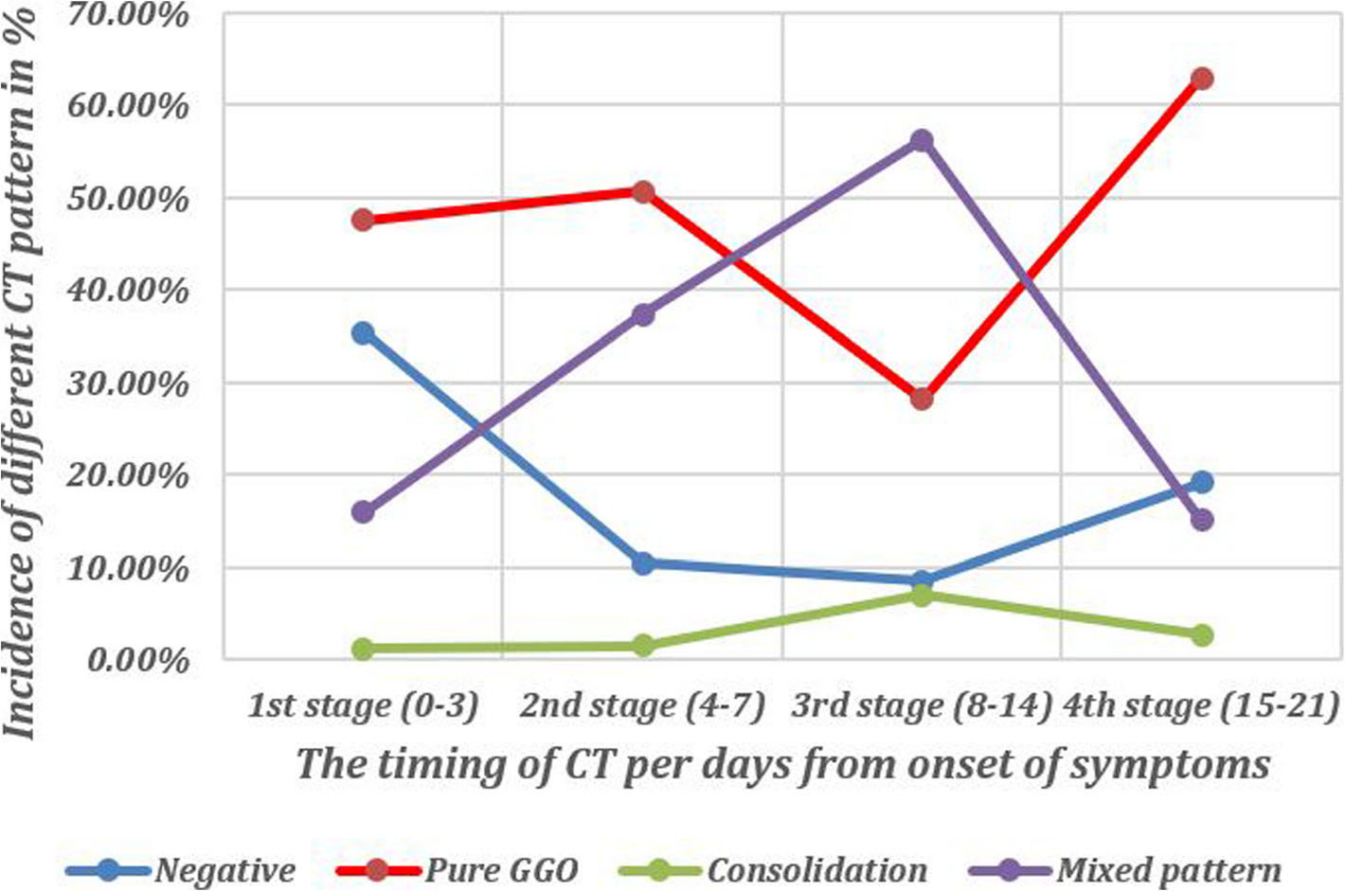
A diagram showing the changes in the incidence of different CT patterns along the different stages of follow-up of cases with COVID-19 infection

**Fig. 4:**
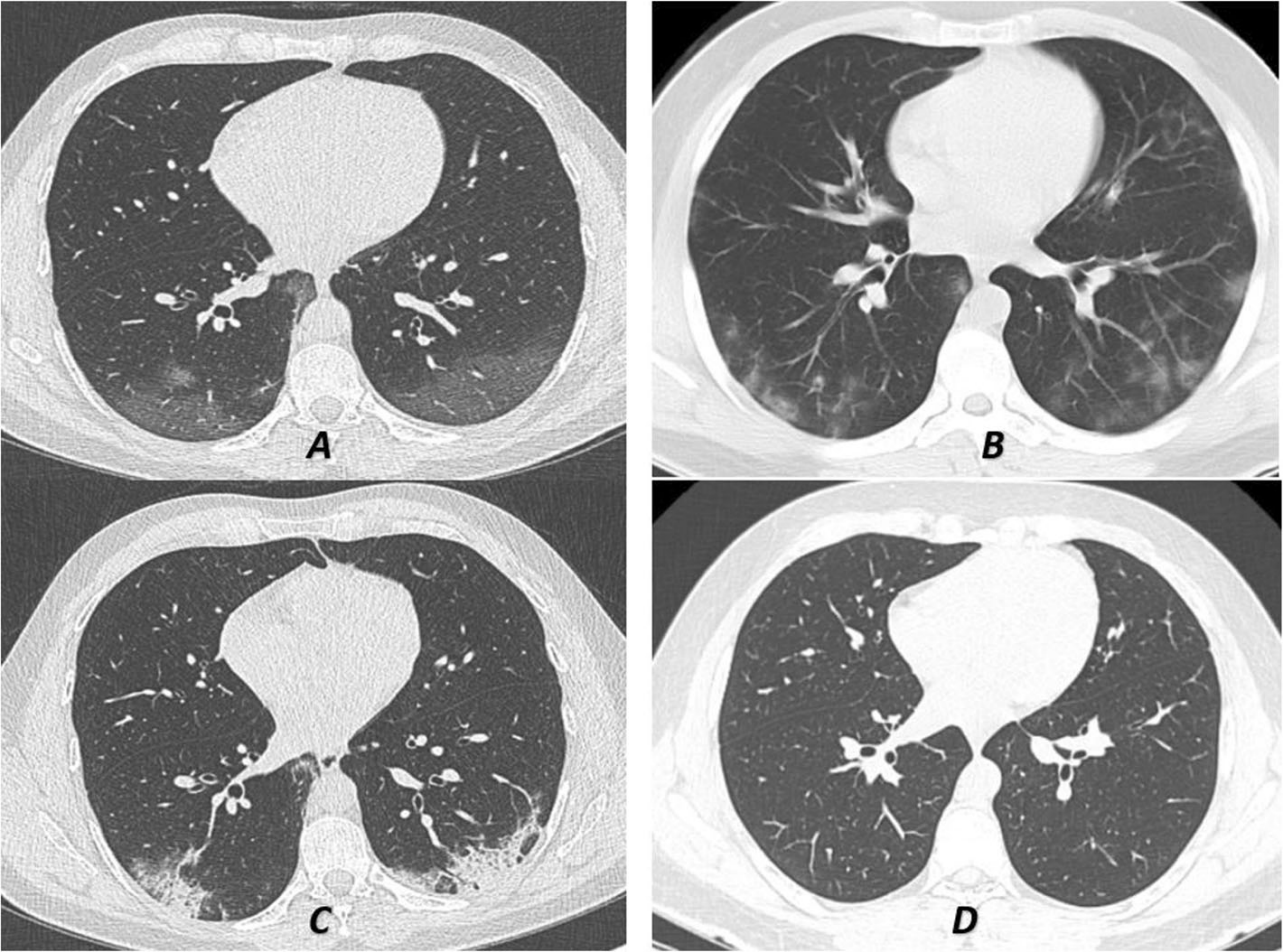
A 27-year-old medical staff who contacted a COVID 19 positive patient and complained of dyspnea and fever. He was diagnosed to have COVID-19 confirmed by a positive RT-PCR test. The 1st CT (lung window) was done 2 days after the onset of the symptoms (**a**) revealed the presence of a bilateral lower lobar posterior and peripheral ill-defined veiling of ground-glass opacity. Follow-up CT (**b**) after 7 days from the onset of symptoms showed multi-lobar subpleural more defined multiple ground-glass patches which denoted disease progression. Peripheral subsegmental consolidative patches with fewer areas of ground-glass opacity were noted in (**c**) which was done after 12 days from the onset of symptoms (stage 3). (**d**) represented CT at the 4th stage after 18 days from the onset of symptoms revealed complete resolution of the previously noted features (negative CT)

The pure GGO pattern was the commonest pattern during the whole course of the disease except in the 3rd stage (8-14 days from the onset of the symptoms) when the mixed pattern predominated. On the other hand, the mixed patterns, either predominant GGO or predominant consolidation, were stationary during different stages except in the 3rd stage when they peaked (56.3%). Moreover, at the 3rd stage, the two subtypes of mixed patterns became significantly different and the predominant consolidation pattern prevailed (Table 2) (Figs. 3 and 4).

### Longitudinal assessment of the involvement of the pulmonary lobes and CT severity score

**Fig. 5:**
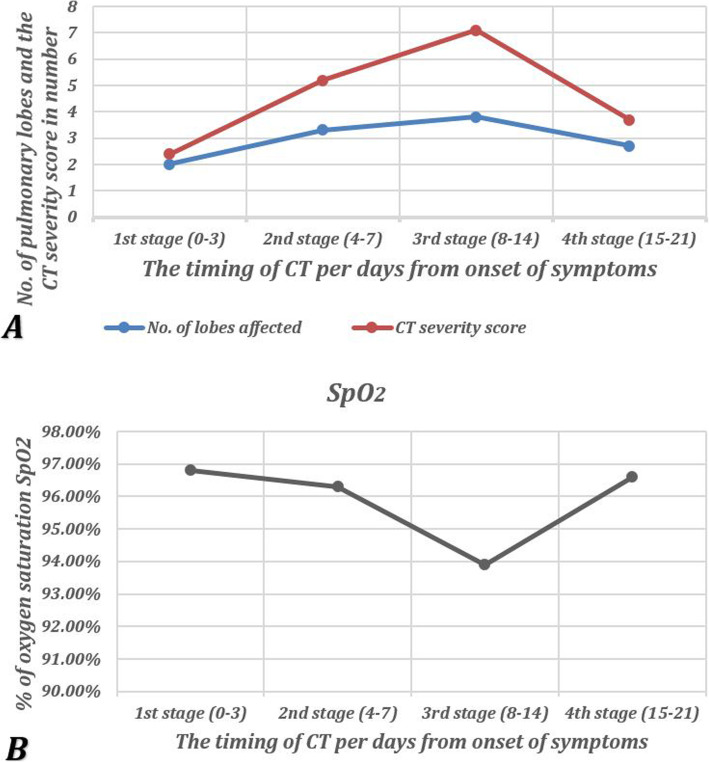
A diagram shows the changes in the numbers of the lobes affected by COVID-19 and CT severity score (**a**) as well as the oxygen saturation (**b**) during different stages of follow-up of cases with COVID-19 infection. As noticed, the more lobes are affected, the higher the CT severity score is, and the lower is the oxygen saturation level

A fewer number of pulmonary lobes were affected in the early stage of COVID 19 pneumonia (2.0 ± 1.9), then more lobes were involved gradually until they reached the peak at the 3rd stage (3.8 ± 1.6) and decreased again in the 4th stage. This relation is directly proportional to the CT severity score which depends on the degree of pulmonary involvement (Table 2) (Fig. 5).

Regarding the complications that were recorded during follow-up, and were the highest at the 3rd stage (19 cases). Pleural effusion was the commonest complication followed by hypoxia. The highest incidence of complications at the 3rd stage was correlated with the incidence of consolidation and the presence of mixed pattern consolidation which can be considered risk factors for complications and signs of disease severity (Tables 2 and 3) (Fig. 2).

**Table 3:** The incidence of consolidation and different CT patterns in complicated cases during different stages of the disease

***Complicated cases***	***1st stage***	***2nd stage***	***3rd stage***	***4th stage***	***Total***
***Number of cases***	3	3	19	3	28
***Consolidation feature***	2 (66.67%)	3 (100%)	18 (94.74%)	3 (100%)	26 (92.86%)
***Pattern***					
***Pure GGO***	1 (33.33%)	0 (0%)	1 (5.26%)	0 (0%)	2 (7.14%)
***Mixed attenuation with predominant GGO***	0 (0%)	1 (33.33%)	3 (15.79%)	0 (0%)	4 (14.29%)
***Mixed attenuation with predominant consolidation***	2 (66.67%)	2 (66.67%)	10 (52.63%)	2 (66.76%)	16 (57.14%)
***Pure consolidation pattern***	0 (0%)	0 (0%)	5 (26.32%)	1 (33.33%)	6 (21.43%)
***Average CT severity score***	12	12.66	10.21	12.33	12

**Fig. 6:**
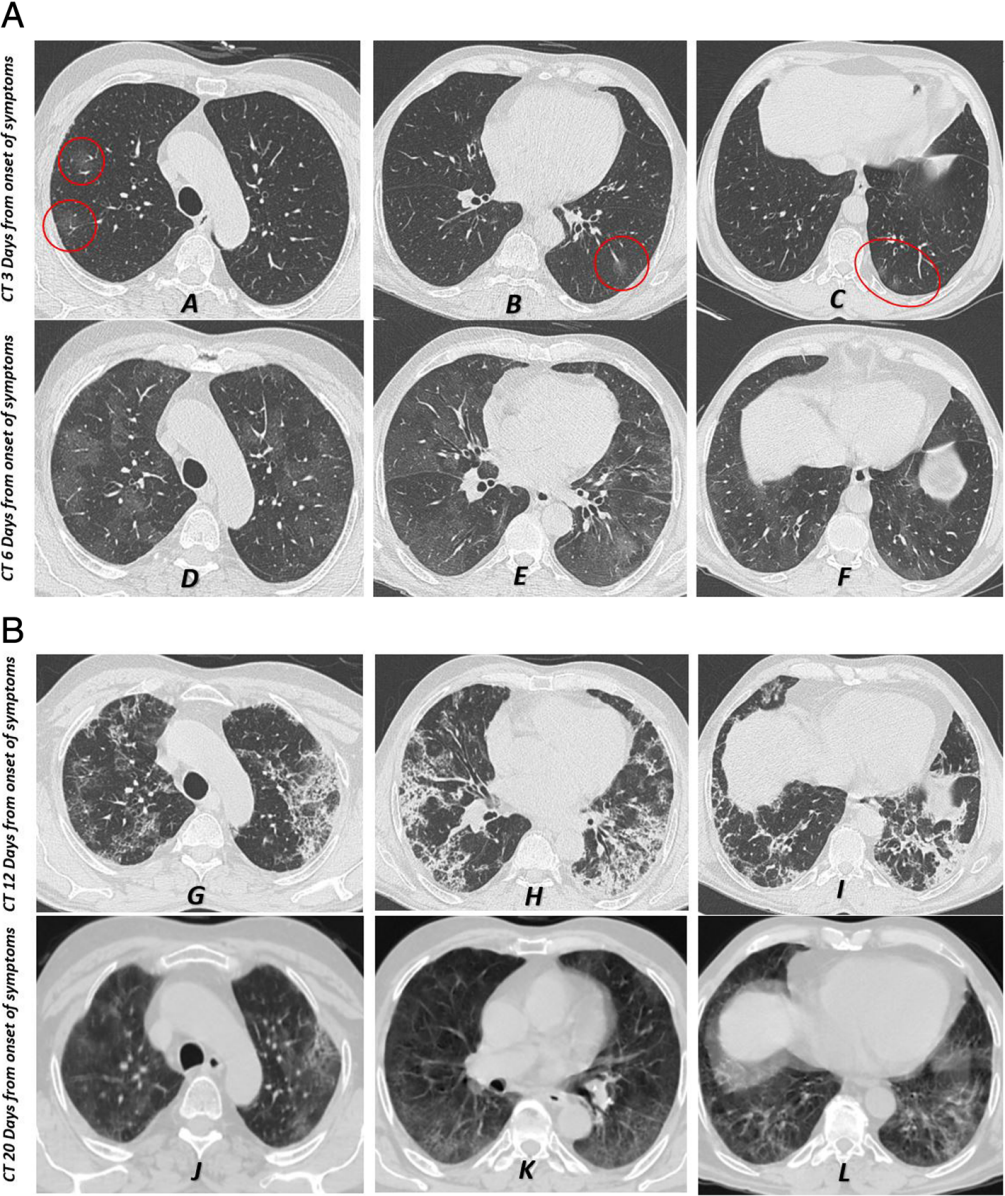
**a**-**l** Axial CT images (lung window) of a male patient 55 years old presented with dyspnea and fever and proved by RT-PCR to have COVID-19 infection. Serial CT scans were done due to the progression of the disease process and the gradual drop in his oxygen saturation. **a**-**c** Represented the CT done during the 1st stage (3 days after the onset of symptoms) and revealed the presence of typical findings of COVID-19 infection which included multiple peripheral rounded ground-glass opacity (red circle). **d**-**f** Represented follow-up CT done at 2nd stage (6 days from the onset of symptoms) and revealed a progressive course in the form of widespread ground-glass opacity and more lobes involved. **g**-**i** Images related to the CT done at the 3rd stage (12 days after the onset of symptoms) showed extensive widespread peripheral consolidation patches and mixed attenuation pattern with predominant consolidation associated with fibrosis. The patient’s oxygen saturation at this stage dropped to 87%; hence, oxygen supply was mandatory and the condition improved. Follow-up CT at the 4th stage (20 days after the onset of symptoms) revealed a regressive course of the CT findings with residual ground-glass opacity (**j**-**l** images)

Regarding the oxygen saturation, the highest values were noticed during the early stage in the disease (96.8 ± 2.2) then gradually declined till the 3rd stage (93.9 ± 3.7) when it reached its lowest value and then grew up again in the late 4th stage. The number of involved lobes and the CT severity score were inversely proportional to the level of the oxygen saturation. In other words, more disease extension, and more lobar affection would increase the CT severity score and decrease the oxygen saturation. This was best detected during the 3rd stage from 8-14 days from the onset of symptoms (Table 2) (Figs. 5 and 6).

Three of our cases needed oxygen therapy during the 1st stage, 3 patients during the 2nd stage, 13 patients during the 3rd stage, only 1 patient in the 4th stage, and this correlates with the pattern of oxygen saturation level during the disease. Six patients required mechanical ventilation; 1 case during the 2nd stage; and 5 cases during the 3rd stage. It was noticed that consolidation features, pure consolidation pattern, and mixed pattern with predominant consolidation were the commonest findings among the patients who required oxygen therapy treatment (Tables 2 and 3).

Finally and from the previous results, we can consider the 1st stage as the initial stage, the 2nd stage as the progressive stage (due to the progression of most of the CT features), the 3rd stage as the peak stage or the critical stage (due to the highest complications and highest CT severity score), and the 4th stage as the resolving stage (due to regression of the CT features and CT severity score). Also, consolidation, mixed attenuation with predominant consolidation, high CT severity score, and low oxygen saturation are considered bad prognostic signs.

## Discussion

This article aims at studying different pulmonary changes that occurred during COVID-19 pneumonia using serial CT scans and exploring more knowledge about the natural history of the disease process. This knowledge is important during the fight against COVID-19 infection which reflects on the morbidity and mortality rates. We studied 125 patients who underwent a total of 293 CT studies (average 2.34 CT study/patient). We divided the CT studies into 4 stages according to the timing of CT after the onset of the symptoms. We assessed different CT features and CT patterns as well as the CT severity score during different stages and recorded the patients’ complications as well as the oxygen saturation during different stages. We found an overall progression of the disease features, extension, and severity over time to reach the peak at the 3rd stage (8-14 days after the onset of symptoms) when the highest incidence of complications and the lowest level of oxygen saturation occurred. This was followed by the regression of these signs during the 4th stage (15-21 days from the onset of symptoms).

Most of the patients enrolled in this study were males (64.8%). Francone M et al. found a higher incidence of COVID-19 between the male gender in their study population (64.6%) and this was very close to our result [13]. Male patients represented 60% of the sample population in the study done by Xiong Y et al. [4]. This was controversial to Salvati L et al. who studied the relation of gender to the incidence, severity, and mortality of COVID-19 and concluded almost equal distribution of the disease between males and females. Although, some difference was found regarding the severity and mortality of the disease being more common in males [14].

Dyspnea and fever were the commonest presenting symptoms found in our sample size (66.4% and 55.2% incidence respectively). Fever was found as one of the most common presenting symptoms [4, 15].

In this study, GGO was the commonest CT feature all through the disease and it showed a gradual rise to reach the peak about 4-7 days from the onset of symptoms, then gradually declined during the last stage. During the last stage, GGO had a higher incidence than the initial stage. This was consistent with Wang Y et al. who found GGO to be the commonest CT features during their study, persisted till the late stage, and became the last finding to resolve [10]. Also, this agreed with Pan F et al. who noticed a progression in the GGOs till the peak stage (9-13 after the onset of symptoms) where most of the GGOs were replaced by consolidations, and this finding was almost similar to our study. Also, they observed extensive GGOs during the absorption stage (> 14 days after the onset of symptoms) as the sequel of consolidation absorption [11].

The incidence of consolidation and crazy paving showed a gradual rise to reach its peak at the 3rd stage (8-14 days after the onset of symptoms) (63.4%) and this was in parallel to the highest incidence of complications as well as the lowest oxygen saturation level at the same stage. Therefore, we can consider the consolidation as a CT sign of severity and prediction of complications. Pan F et al.’ study agreed with this, as they noticed consolidation changes in 90% of the patients at the 3rd stage which was also the peak [11]. Liang T et al. also showed a progressive increase in the incidence of consolidation that resolved gradually after 2 weeks from the onset of symptoms [16]. Wang Y et al. hypothesized that consolidation during this stage was due to organizing pneumonia which had the potential to progress to fibrosis and mandated the increase in steroid therapy [10]. Liu et al. studied 73 patients and found that consolidation was the commonest CT feature in patients with severe infection [17]. Liu N et al. also found that consolidation was the commonest feature during the follow-up study (81%) and gradually decreased during further follow-up [18].

Fibrosis was not seen in all cases particularly in the early stages. Yet, its incidence increased in the late stage (4th stage) 15-21 days from the onset of the symptoms, when most of the features, CT severity, and oxygen saturation improved. Therefore, we could consider the appearance of fibrosis as a sign of healing and resolution of the disease. This was consistent with Wang Y et al. and Liang et al. who found a higher incidence of fibrosis in the late stage in their studies and also confirmed it as a sign of resolution of the disease process [10, 16]. Xiong Y et al. studied 42 patients with initial and follow-up CT done at 4.5 and 11.6 days respectively after the onset of symptoms and concluded that the incidence of fibrosis almost doubled from 36% in the initial CT to 74% in the follow-up CT [4].

According to our study, there was a gradual progression of the pulmonary opacities, an increase in crazy paving and consolidation, and consequently mixed attenuation pattern through the course of the disease to reach the peak at the 3rd stage and then dropped in the 4th stage. Moreover, mixed attenuation with predominant consolidation was commoner than mixed attenuation pattern with predominant GGO during the 3rd stage. These were keeping with other few studies [10, 13, 16]. Also in complicated cases, we noticed a higher incidence of mixed attenuation patterns with predominant consolidation during different stages of the disease rendering it a CT sign of severity.

Different CT scoring systems were used to assess the disease severity, some of them depended on the assessment of the disease extension whatever the pattern of the disease, while others depended on both the disease extension as well as the pattern of CT features [10, 11, 16, 19, 20]. We used a simple CT scoring similar to the one used by Francone M and Pan F et al. [11, 13] which depended on the extension of the disease. It was simple, rapid, and had less inter-observer variability.

In our study, we noticed more lobar affection, and hence worse CT severity score, during the disease which peaked at the 3rd stage (8-14 days from the onset of the symptoms). Xiong et al. compared the affected lobes during the initial and follow-up CT (average 7 days interval) and found a significant increase in the number of the affected lobes from 3.7 ± 1.6 in initial CT to 4.4 ± 1.2 in follow-up CT [4]. Our results agreed with Pan F et al. who found the peak of CT score and several lobes involved in stage 3 (9-13 days from the onset of symptoms) and the peak CT score was 7 ± 4 compared to 7.1 ± 5.1 in our study [11]. Also, Liang T et al. noticed the peak of CT score occurred at days 11-14 during follow-up. However, the score was 5.3 ± 3.3 which was lower than our study [16].

Furthermore, we detected the highest CT severity score in complicated cases during the 2nd and 3rd stages (12.66 and 10.21 respectively) and this was in line with Liu N et al. who found a higher CT score in patients with severe COVID-19 infection compared to patients with moderate disease form and the peak CT score in severe cases was 12.1 during the second follow-up (average 13 days after the onset of symptoms) [18].

Oxygen saturation was considered one of the main important parameters that indicated disease severity and progression. We noticed an inverse relationship between the CT severity and the level of oxygen saturation, as the higher the CT severity score was, the lower the level of oxygen saturation. The lowest level of oxygen saturation noticed in our study was at the 3rd stage and this could be explained by the highest incidence of mixed attenuation pattern with predominant consolidation at this stage. Moreover, the progressive development of fibrosis came in parallel with more disease extension, higher numbers of lobar involvement, and higher CT severity score. This was compatible with Wang K et al. who studied 114 confirmed cases with COVID-19 pneumonia and found an inverse correlation between CT and SpO_2_ [21]. Also, Dia H et al. found a drop in SpO_2_ level with the progression of the disease and also noticed that more diffuse parenchymal involvement, consolidation, and fibrosis explained the severity of pulmonary dysfunction [22].

### Limitation

The limitations of our study were being a retrospective. Hence, all patients underwent their CT studies according to the clinical condition and not at a regular fixed interval. We did not divide our patients into subgroups depending on the clinical severity which required more researches to acquire more data about the disease progression between different clinical groups. Moreover, long-duration follow-up is needed for more understanding of long term effects and pulmonary changes.

## Conclusion

It is important during our fight against COVID-19 infection to know the nature of the disease and the changes that occur during different stages of the disease to reduce patients’ morbidity and mortality. It is important to know that COVID-19 pneumonia usually shows gradual radiological progression features to reach a peak in the 2nd week after the onset of the symptoms. The second week is the most critical time during the disease course and has the highest CT severity score, the highest incidence of complications, and the lowest oxygen saturation level. This is followed by a slow regression during the 3rd week. Also, consolidation, mixed attenuation with predominant consolidation pattern, and high CT severity score are considered CT signs of severity and bad prognosis.

## Data Availability

Available on request with the corresponding author.

## Abbreviations

COVID-19: Coronavirus disease 2019
ACE2: Angiotensin-converting enzyme 2
GGO: Ground-glass opacity
CT: Computed tomography
PCR: Polymerase chain reaction
RT-PCR: Reverse transcription-polymerase chain reaction
SpO_2_: Oxygen saturation
